# Cultivating a Therapeutic Compassionate Relationship: The 3S Approach

**DOI:** 10.25122/jml-2019-0045

**Published:** 2019

**Authors:** George Samoutis, Sophronia Samouti, Pansemni A Aristodemou

**Affiliations:** 1. Primary Care and Population Health, University of Nicosia Medical School, Nicosia, Cyprus; 2. International Institute for Compassionate Care - Youth Unit, Nicosia, Cyprus; 3. International Institute for Compassionate Care - Academic Commitee, Nicosia Cyprus

**Keywords:** Compassion, Compassionate care, Chronic disease management

## Abstract

In the last decade, a plethora of healthcare research and literature was produced and, indeed, confirms the absolute need to cultivate a therapeutic and compassionate relationship between carer and patient/family, especially in the face of a long-term and /or life-threatening condition. We introduce the 3S model as an approach to cultivate a therapeutic relationship between the carer and the patient/family. It is based on some fundamental traditional skills which may be innate for some but may need to be awakened and cultivated for others, all for the benefit of each of the members of the involved triad: patients, family, and healthcare professionals.

The 3S approach aids in developing a therapeutic relationship that involves compassion and can be easily applied with significant results, especially in the context of chronic disease management. However, more research is needed to quantify the impact of this 3S approach on the therapeutic relationship and chronic disease management.

## Introduction

The definition of a therapeutic relationship between carer and patient/family is defined as a helping relationship that is based on mutual trust and respect, the nurturing of faith and hope, being sensitive to self and others, and assisting with the gratification of your patient’s physical, emotional, and spiritual needs through your knowledge and skills [1,2].

From Classical Greek days, Aristotle (384-322BC) described compassion as one of the five virtues needed in order for a person to flourish and be happy, and concluded on its eudemonistic origins i.e. that it arises from the recognition of general vulnerability (knowing that fate can strike oneself just like it has my brother) [3]. Compassion, literally meaning suffering together with someone (Latin “com”=together, “passio”=suffering), describes a deep awareness of the suffering of another, coupled with a desire to relieve it [4]. Today, in the international literature, bringing care to healthcare is described in terms of a sympathetic presence and person-centeredness, the respectful deference and acceptance of the other, and a positive connectedness which is intrinsic to the shaping of a therapeutic relationship between carer and patient/family [5].

The role of compassionate care in our current healthcare systems is considered by all stakeholders (patients, families, clinicians, and policymakers) as an important element of high-quality care and helps to build a therapeutic relationship [6-8].

However, recent evidence showed that even in developed countries, healthcare sometimes fails at a fundamental level, and lack of compassionate care and suboptimal care is a pragmatic situation in an array of organizations [9]. There is a strong suggestion that compassionate care is the missing link between care that is based on best scientific evidence and a good, intuitive, heartfelt career-patient relationship: together, best quality healthcare is achievable [10].

Moreover, a scoping review of the healthcare literature has concluded that there is limited empirical understanding of compassion in healthcare and highlighted the lack of patient and family voices in compassion research. It also suggests that a deeper understanding of the key behaviors and attitudes that lead to improved patient-reported outcomes through compassionate care is absolutely necessary [11].

Within this paper, we propose the 3S approach (Symponesis, Symporefsis, Synchoresis) as a way of improving the patient-centered, therapeutic relationship, and more specifically, we refer to palliative care as an example of implementing the aforementioned model.

## Discussion

### The proposed 3S approach to embed a therapeutic relationship

**Figure 1:**
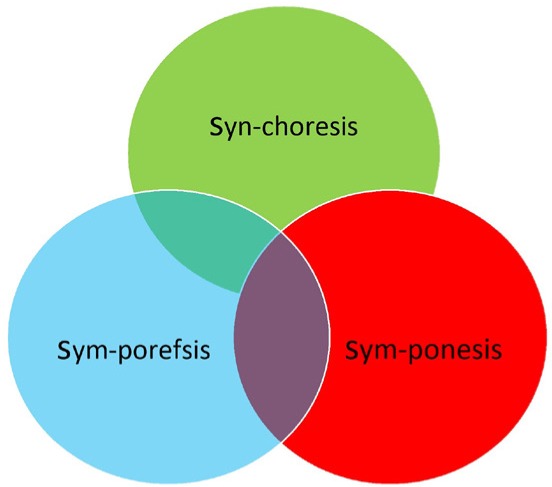
The 3S approach model for compassionate care.

The model follows the 3S approach based on three fundamental skills/virtues: Synchoresis, Symporefsis, and Symponesis (Figure 1).

Etymologically, all the three words in our model come from the Greek “syn”, συν=together, (phonetically becomes “sym” if the following word starts with p):

• Symponesis (greek “συν” + “πονέω”, suffer with, take part in toils of other) [12],
• Symporefsis (greek “συν” + “πορεύομαι”, walk together, accompany) [12],
• Synchoresis (greek “συν” + “χωρέω”, come to the same place, concede, agree, forgive) [12], as a way of implementing a therapeutic relationship, more specifically in relation to long-term diseases care and palliative care, especially in end-of-life care.

### Symponesis: “Weep with those who weep” [11].

Although cultural expressions of “symponesis” may differ [14], “symponesis” lies at the heart of most religious, ethical and spiritual traditions, calling us always to treat others as we wish to be treated ourselves.

**Table 1:** The golden rule.

The golden rule:	
**Buddhism:**	“Hurt not others in ways that you would find hurtful.” [15]
**Christianity:**	“Do unto others as you would have them do unto you.” [16]
**Confucianism:**	“Do not do unto others what you do nt want done unto you.” [17]
**Hinduism:**	“This is the sum of duty: do not do to others what would cause pain if done to you.” [18]

Yet, applying “Symponesis” requires effective interpersonal skills, attentive listening, presence, recognition of the value of small caring gestures, promising never to abandon, and physical alleviation of the patient’s suffering, thus strengthening the mystery of healing that is enacted in the therapeutic relationship that is formed.

An array of initiatives helped inspire healthcare organizations to bring back “symponesis,” claiming that it is equal to a form of consistent altruism specifically visible by practicing the above golden rule [19-21].

“Symponesis” in nursing practice comprises the enactment of personal and professional values through behaviors that demonstrate the emotional and spiritual dimension of caring about another person as well as the practical dimension of caring for them in a way to recognize and alleviate their suffering [22].

Of absolute significance in this ‘ceremony’ of “symponesis,” there must be recognition, acceptance, and respect for the uniqueness of the ‘person’ in that patient in need. Indeed, this is being more than ‘just kind’.

Implicated in this is the capacity to empathise, i.e. the ability to supportively communicate a sensitive awareness and affirmation of another person’s feelings and the unique meanings attached to them [23], something that is central to the development of the therapeutic relationship between the patient and the carer, and an integral part of healing [24-26]. And this may form the basis of education tools for implementing compassion into healthcare [27].

### Symporefsis: “A pain shared is a pain halved” [28].

Symporefsis (from “syn” =together and “poreia” =walk, Greek) translates into walking with/accompanying. The mystery of togetherness, or of the “com” prefix in Latin and the “syn” (or sym if the following word starts with “p” in Greek), is vital to the fulfillment of all human values. “No man is an island,” writes poet John Donne, and this implies the integral necessity for societal interlinking [29].

There is, indeed, a healing power in togetherness on many levels, both practical and psychological, but also the positive energy from the carers can be literally therapeutic, as described in the miracle of healing the paralytic [30] where Christ heals him because of the faith of those who carried him (down the roof)! In other words, the value of being with a patient can confer a wealth of positive results, both to the patient and his caregiver.

Halving someone’s suffering just by sharing it with him, by suffering together with him is the great mystery of kenotic love, a prime asset of compassionate care. What is meant by kenotic love may not be obvious to all and needs to be clarified: kenosis is the mystery of self-emptying [31], of extinguishing all that comes from the self, the ego and simply abandoning oneself to the will of the other, whomever he may be. In the case of compassionate healthcare, where the art of walking by the side of a patient who is in need, he, who decides to undertake this accompaniment (symporefsis) must be equipped with absolute kenosis. Moreover, in order to achieve this symponesis, (syn=together, ponos=suffer, Greek), one needs to cultivate the art of ‘synchoresis’.

### Synchoresis: “If you want to go fast, go alone. If you want to go far, go together.” [32].

Synchoresis (“syn” =together, “choros” =space, place, Greek) refers to being together with the patient in the same ‘place’, wherever that may be physically or spiritually (in space or in spirit). The “synchoresis”, which is the literal translation of being together, just as “symponesis” is the literal paraphrase of hurting/suffering together, and “symporefsis” of travelling together, can only be achieved when we “enlarge” our hearts to contain all other irrelevant on their socioeconomic, race and educational background enabling a therapeutic relationship [33]. Cultural competence is extremely important in terms of accepting the other and manage to embrace him/her in a therapeutic relationship.

### The case of palliative care

Palliative care is an approach that improves the quality of life of patients and their families facing the problems associated with a life-threatening illness, through the prevention and relief of suffering by means of early identification and impeccable assessment and treatment of pain and other problems, physical, psychosocial and spiritual [34].

Yet, compassionate palliative care focus on the acknowledgment of the great losses that accompany a life-threatening disease coinciding with excellent communication skills, competence in practice, courage, clarity, and confidence in a relationship as well as a clear commitment to the person in need, be it the patient or his family.

Existentially, one and his family face an ultimate crisis when being diagnosed with a life-threatening condition. Significant advances are being made in alleviating most of the physical symptoms that man can suffer from, and for the psychological and social problems that he and his family may face as well. The spiritual, existential questions that may arise, however, may not be so ‘easy’ to solve or handle, except by the cultivation and application of Symponesis, Synchoresis, Symporefsis, namely the 3S approach. The ‘just being there’ (despite all), which is often quoted in modern literature, is nothing less than the art of selfless, compassionate caring that we are desperately in need of today. We live in the days where modern man is forevermore isolated from his fellow humans, closed up in his individualistic search for a meaning of his life, individualistic and antagonistic strife to materially succeed (the rat race), that often leads to a great vacuum.

This is where the reaching out by even stranger professionals or trained volunteers, community workers willing to take on the burden of the other, whomever he may be, comes to throw down all rites and rubrics about self-preservation, survival, even self-care. Compassionate care encompasses the other more than oneself in a total and final sense.

### Cultivating compassion in Youth

The cultivation of the proposed 3S approach needs to start early in education, especially in the healthcare professionals’ undergraduate courses. The International Institute for Compassionate Care (IICC) and others have been providing relevant under and postgraduate courses have shown that compassion can be taught and cultivated [35]. The future healthcare professionals also need to be exposed early on in their education on compassionate care. Thus, the IICC youth team has developed a short course (blended learning, 4 hours online, and 2 hours onsite) for the healthcare school students candidates (medicine, nursing, and so forth). The key topics include the definition of compassion, compassion fatigue, a therapeutic relationship, and patient-centeredness. The evaluation of the impact of this course is expected.

## Conclusions

There is much literature on the subject of compassion and empathy in healthcare and how much they are needed as part of quality care; many models are being developed for reintroducing it in the teaching curricula of healthcare professionals [27, 35,36]. Thankfully, it now seems that we are trying to return to some old community values that our grandparents were practicing routinely. One may remember having a grandparent home to look after until the end, something our parents did, not so long ago; yet today, we have no time, no space, no spirit to take on someone in need (even if they are within our own family). Nevertheless, is it because there is genuinely no space in our house or in our timetable, or is it just because there is no space in our hearts?

By relearning old basic rules about ‘loving your neighbor like yourself’ [37], and ‘carrying each other’s burdens’ [38], we can achieve the mystery of kenotic love of the other by losing ourself in his will and accepting him non-judgingly. Thus, we can indeed be with him in every space he is (synchoresis), we can selflessly accompany him every step of his journey (symporefsi), and ultimately we can suffer with him (symponesis) by just totally and selflessly empathizing with him.

The training of future healthcare professionals on compassionate care principles even before they start their undergraduate training is of paramount importance. If we would like to change the culture in our healthcare systems towards a more compassionate and patient-centered one, we need to invest in the youth.

## Conflict of Interest

The authors confirm that there are no conflicts of interest.
